# Cerebral microbleeds in patients with COVID-19: is there an inevitable connection?

**DOI:** 10.1093/braincomms/fcae236

**Published:** 2024-07-19

**Authors:** Yuchang Wang, Yuetao Hu, Ruichen Zhao, Qi Wang, Jiarui Xu, Jiangyuan Yuan, Shiying Dong, Mingqi Liu, Chenrui Wu, Rongcai Jiang

**Affiliations:** Department of Neurosurgery, Tianjin Neurological Institute, State Key Laboratory of Experimental Hematology, Key Laboratory of Post-Neuroinjury Neurorepair and Regeneration in Central Nervous System Tianjin & Ministry of Education, Tianjin Medical University General Hospital, Tianjin 300052, China; Department of Neurosurgery, Tianjin Neurological Institute, State Key Laboratory of Experimental Hematology, Key Laboratory of Post-Neuroinjury Neurorepair and Regeneration in Central Nervous System Tianjin & Ministry of Education, Tianjin Medical University General Hospital, Tianjin 300052, China; Department of Neurosurgery, Tianjin Neurological Institute, State Key Laboratory of Experimental Hematology, Key Laboratory of Post-Neuroinjury Neurorepair and Regeneration in Central Nervous System Tianjin & Ministry of Education, Tianjin Medical University General Hospital, Tianjin 300052, China; Department of Neurosurgery, Tianjin Neurological Institute, State Key Laboratory of Experimental Hematology, Key Laboratory of Post-Neuroinjury Neurorepair and Regeneration in Central Nervous System Tianjin & Ministry of Education, Tianjin Medical University General Hospital, Tianjin 300052, China; Department of Neurosurgery, Tianjin Neurological Institute, State Key Laboratory of Experimental Hematology, Key Laboratory of Post-Neuroinjury Neurorepair and Regeneration in Central Nervous System Tianjin & Ministry of Education, Tianjin Medical University General Hospital, Tianjin 300052, China; Department of Neurosurgery, Tianjin Neurological Institute, State Key Laboratory of Experimental Hematology, Key Laboratory of Post-Neuroinjury Neurorepair and Regeneration in Central Nervous System Tianjin & Ministry of Education, Tianjin Medical University General Hospital, Tianjin 300052, China; Department of Neurosurgery, Tianjin Neurological Institute, State Key Laboratory of Experimental Hematology, Key Laboratory of Post-Neuroinjury Neurorepair and Regeneration in Central Nervous System Tianjin & Ministry of Education, Tianjin Medical University General Hospital, Tianjin 300052, China; Department of Neurosurgery, Tianjin Neurological Institute, State Key Laboratory of Experimental Hematology, Key Laboratory of Post-Neuroinjury Neurorepair and Regeneration in Central Nervous System Tianjin & Ministry of Education, Tianjin Medical University General Hospital, Tianjin 300052, China; Department of Neurosurgery, Tianjin Neurological Institute, State Key Laboratory of Experimental Hematology, Key Laboratory of Post-Neuroinjury Neurorepair and Regeneration in Central Nervous System Tianjin & Ministry of Education, Tianjin Medical University General Hospital, Tianjin 300052, China; Department of Neurosurgery, Tianjin Neurological Institute, State Key Laboratory of Experimental Hematology, Key Laboratory of Post-Neuroinjury Neurorepair and Regeneration in Central Nervous System Tianjin & Ministry of Education, Tianjin Medical University General Hospital, Tianjin 300052, China

**Keywords:** cerebral microbleeds, COVID-19, pathogenesis, brain pathology

## Abstract

The COVID-19 pandemic has underscored the critical interplay between systemic infections and neurological complications, notably cerebral microbleeds. This comprehensive review meticulously aggregates and analyses current evidence on cerebral microbleeds’ prevalence, pathophysiological underpinnings and clinical implications within COVID-19 cohorts. Our findings reveal a pronounced correlation between cerebral microbleeds and increased severity of COVID-19, emphasizing the role of direct viral effects, inflammatory responses and coagulation disturbances. The documented association between cerebral microbleeds and elevated risks of morbidity and mortality necessitates enhanced neurological surveillance in managing COVID-19 patients. Although variability in study methodologies presents challenges, the cumulative evidence substantiates cerebral microbleeds as a critical illness manifestation rather than mere coincidence. This review calls for harmonization in research methodologies to refine our understanding and guide targeted interventions. Prioritizing the detection and study of neurological outcomes, such as cerebral microbleeds, is imperative for bolstering pandemic response strategies and mitigating the long-term neurological impact on survivors.

## Teaser

Wang *et al*. report a notable association between cerebral microbleeds (CMBs) and severe coronavirus disease 2019 (COVID-19), emphasizing the role of inflammatory responses and vascular disruptions. The review highlights the need for standardized research methodologies to better understand CMBs’ clinical impact and suggests integrating neurological monitoring into COVID-19 management strategies.

## Graphical Abstract

**Graphical abstract:**
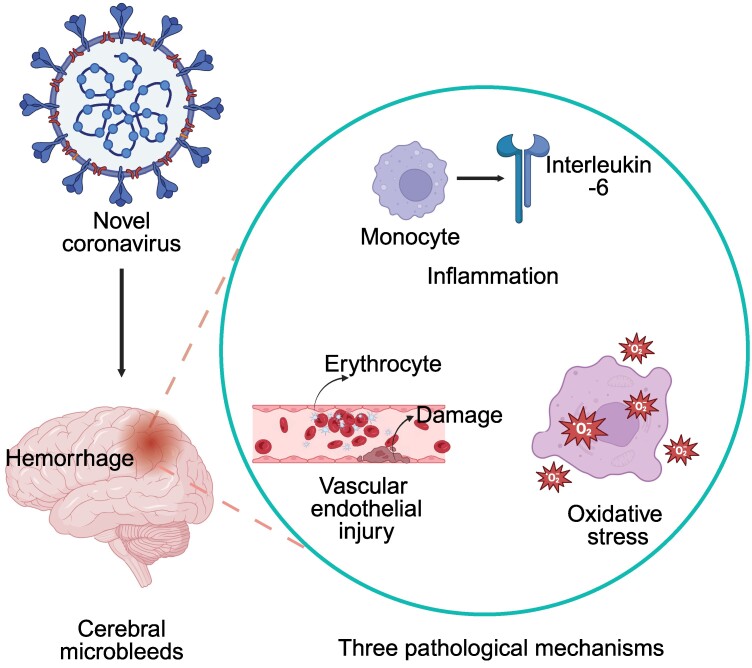

## Introduction

The aetiological culprit of the emergent respiratory infection known as COVID-19 is the severe acute respiratory syndrome coronavirus 2, which first surfaced in China’s Hubei province in late 2019.^1^ This novel coronavirus has since precipitated a global pandemic characterized by widespread atypical severe pneumonia. The World Health Organization has designated the condition as COVID-19, reflecting its status as a novel disease entity recognized in 2019.^2^ Severe acute respiratory syndrome coronavirus-2 (SARS-CoV-2) exhibits high pathogenicity, with potential to infect diverse cell types and tissues.^3^ While primarily presenting as a respiratory illness, an expanding body of evidence recognizes neurological complications as a significant aspect of COVID-19.^4^ Numerous studies have documented a spectrum of neurological sequelae associated with the disease.^5^

Cerebral microbleeds (CMBs), appearing as hypointense foci on T2*-weighted and susceptibility-weighted magnetic resonance imaging (MRI) sequences, are neuroimaging findings often associated with cerebrovascular disease, dementia and the aging process.^6-8^ Since the incorporation of advanced MRI techniques in the 1990s, CMBs have gained prominence as clinically relevant entities.^9^ The detection of these lesions has become more frequent, highlighting their importance in the neurological landscape. Histologically, CMBs are characterized by small accumulations of macrophages engorged with iron-rich haeme, often found in conjunction with aberrant blood vessels.^10^ Acute microhaemorrhages can be discerned by the presence of intact or lysed red blood cells in standard haematoxylin and eosin stained sections. Beyond 24 h post-extravasation, in the subacute phase, lysed red blood cells give way to hemosiderin or haematoidin as blood-breakdown products, subsequently engulfed by macrophages.^7^ There have been several remarkable developments in the study of CMBs in recent years. Enhancements in MRI technology, both in software and hardware, have markedly improved sensitivity, resulting in a higher rate of CMBs detection and a refinement of the criteria used for their identification.^11^

In critically ill patients, particularly those suffering from acute respiratory distress or disseminated intravascular coagulation, CMBs are commonly observed in the subcortical white matter and the corpus callosum.^8,12,13^ A systematic review revealed that 58.7% of the MRI scans positive for CMBs post-COVID-19 identified these lesions in the corpus callosum or adjacent cortical areas.^14^ One possible explanation is that in severe COVID-19, the hyperinflammatory cascade, notably characterized by a cytokine storm, leads to endothelial dysfunction and coagulopathy.^15^ This pathological milieu may predispose the corpus callosum, particularly its splenium region characterized by an abundance of cytokine and glutamate receptors, to cerebral microhaemorrhages.^16^ The presence of CMBs in patients with COVID-19 is indicative of a grave prognosis, correlating with critical illness, diminished functional outcomes and increased mortality rates.^17-19^ Furthermore, a prospective study indicated that individuals with CMBs had a nearly threefold increased risk of experiencing a subsequent stroke that was either disabling or fatal, compared to those without such lesions.^20^

Several hypotheses have emerged to explain the pathogenesis of COVID-19-related neurological disorders, with CMBs posited as a consequential late-stage manifestation of severe hypoxaemia in affected patients.^21-23^ Although the precise pathophysiological mechanisms remain elusive, one prevailing theory suggests that SARS-CoV-2-inflicted endothelial damage—mediated by the virus’s interaction with angiotensin-converting enzyme 2 (ACE2)—leads to a cascade of events. This interaction may disrupt the renin-angiotensin system, alter lipid metabolism and activate the ceramide pathway.^24^ Such endothelial compromise might result in reactive oxygen species production, vasoconstriction and subsequent hypoxia, culminating in the formation of CMBs. These pathophysiological events are believed to exacerbate the severity of COVID-19, potentially escalating to fatal outcomes.^23^

The aetiology of COVID-19-related nervous system disorders is multifaceted, with CMBs emerging as a notable concern.^25^ It remains to be seen whether the CMBs in COVID-19 are caused directly by SARS-COV-2 attacking neurons or blood vessels,^26^ indirectly by sequelae of critical illness^27^ or entirely by coincidence. Our forthcoming presentation will encompass the epidemiology of CMBs in COVID-19 patients, their clinical repercussions and significance, while also delving into the pathological underpinnings of microbleeds in the context of this viral infection.

## Search strategy and selection criteria

Electronic literature search was performed using PubMed (National Center for Biotechnology Information, USA) database, repositories such as Semantic Scholar (Allen Institute for Artificial Intelligence, USA), Europe PubMed Central (EMBL-EBI, UK), JSTOR (USA) and the Google Scholar search engine (USA). Various combinations of the following keywords were employed for searching and screening of relevant information: CMBs, COVID-19, SARS-CoV-2, neurological diseases, neuroinflammation, pathogenesis, brain pathology and neuroimaging.

## Epidemiology

### CMBs in COVID-19 patients: a relatively high incidence

Data analysis from 361 patients revealed that brain abnormalities occurred in approximately one-third of the cohort.^28^ The most prevalent finding was cerebral white matter hyperintensities, with CMBs, haemorrhages and infarcts also frequently observed.^22,28^ According to different backgrounds and clinical conditions, the incidence of CMBs in patients with COVID-19 ranged from 21.74% to 63.64% (**Table 1**). In these studies, it is noteworthy that the proportion of males within the CMBs populations was found to be relatively high, with percentages of 71%, 56% and 84%, respectively.^23,29,30^ However, the limited number of subjects in these studies poses a constraint, necessitating further research to substantiate and clarify the gender effects observed. The incidence of CMBs in COVID-19 patients is influenced by different backgrounds and clinical conditions, as highlighted by the reported incidence rates ranging widely across studies. It is important to note that these figures do not represent a definitive incidence rate, since a global, systematic screening of the population has not been conducted. The heterogeneity of these results suggests that various factors, such as patient demographics, pre-existing health conditions and the severity of COVID-19 infection, are likely to play a significant role in the observed prevalence of CMBs. This underlines the need for more widespread and uniform data collection methods to accurately determine the incidence rates of CMBs in the context of COVID-19.

**Table 1:** Raito of CMBs with COVID-19

Study	Country	No. of COVID-19	No. of CMBs/COVID-19	Title of thesis
Stéphane Kremer (multicentre experiment)	France	37	9 (24.32%)	(8)
Luke Dixon (single centre experiment)	UK	28	10 (35.71%)	(13)
Shaahank Agarwal (multicentre experiment)	UK	115	25 (21.74%)	(19)
Alireza Radmanesh (single centre experiment)	UK	11	7 (63.64%)	(21)
Francois Lersy (single centre experiment)	France	80	19 (23.75%)	(23)
Angela Napolitano (single centre experiment)	Italy	63	14 (22.22%)	(29)
Henriikka Ollila (single centre experiment)	Finland	161	48 (29.81%)	(30)
François Lersy (single centre experiment)	France	31	9 (29.03%)	(109)

Abbreviations: No. of COVID-19 = number of patients with COVID-19; No. of CMBs/COVID-19 = number of CMBs in patients with COVID-19.

While initial research on COVID-19-related CMBs primarily concentrated on critically ill patients, recent studies have expanded the scope to include non-hospitalized COVID-19 patients. In a study that assessed organ-specific function in individuals following mild to moderate SARS-CoV-2 infection, the presence and number of CMBs and white matter hyperintensities, the most common hallmarks of cerebral small vessel disease were comparable between participants recovered from mild to moderate COVID-19 and controls.^31^ Notably, Sagar *et al*. demonstrated a significant incidence of CMBs in non-hospitalized COVID-19 patients, with an odds ratio of 2.66 for the presence of CMBs compared to non-infected controls, even after adjusting for confounding factors such as age, sex and comorbidities.^32^ These findings highlight the importance of monitoring neurological complications in all COVID-19 patients, regardless of their hospitalization status.

In patients with cerebrovascular disease, CMBs serve as indicators of the severity of underlying vascular pathology. The presence of CMBs elevates the risk of subsequent stroke,^33,34^ dementia^35^ and mortality.^36^ Among patients experiencing their first ischaemic stroke, the prevalence of CMBs is approximately 23%; this figure rises to about 44% in those with recurrent ischaemic strokes, and to 60% in cases of intracerebral haemorrhage.^37,38^ With advancements in and broader access to imaging technologies, the detection rates of CMBs are anticipated to rise significantly in the near future. The escalating prevalence of CMBs with the severity and recurrence of cerebrovascular events highlights their value as a prognostic tool, necessitating their integration into clinical risk assessment and management. Moreover, the expected increase in CMBs detection through improved imaging technology underscores the need for updated clinical guidelines to address the evolving landscape of cerebrovascular disease diagnosis and treatment.

### COVID-19 may be a risk factor for CMBs

CMBs are associated with a spectrum of cerebrovascular conditions such as stroke, cerebral amyloid angiopathy (CAA) and hypertensive vascular disease.^39-41^ Additionally, CMBs have been identified in a range of other medical contexts, including sepsis, Parkinson’s disease, chronic obstructive pulmonary disease, traumatic brain injury and even among the asymptomatic elderly.^42,43^

Age is independently associated with the presence of CMBs and remains a relevant factor in the clinical status of COVID-19 patients.^30,44^ A clinical investigation revealed a significant association between the emergence of intracerebral haemorrhagic lesions in individuals with COVID-19 and a deterioration in respiratory function, neurological status and overall physiological parameters.^8^ Moreover, research indicates that CMBs prevalence escalates with advancing age among adults free from stroke, and this prevalence correlates strongly with increasing age.^43^ However, it is important to emphasize that CMBs are not considered a normal aspect of aging. The age-related rise in CMBs is likely attributable, in part, to the progressive accumulation of vascular risk factors over time.^30^ CMBs have been associated with various clinical conditions, such as diffuse axonal injury, exposure to high altitudes and brain radiation therapy. Nonetheless, CAA and hypertensive vasculopathy remain the predominant risk factors.^43^ CAA arises from the deposition of β-amyloid in the walls of cortical and leptomeningeal vessels, while hypertensive vasculopathy, related to chronic high blood pressure, is characterized by hyaline-like changes in deep, penetrating small arteries.^41^ CMBs influenced by these conditions typically affect the basal ganglia, thalamus and periventricular white matter.^45^ Given that both CAA and hypertensive vasculopathy are age-related, they may coexist with variable severity in the same individuals.^46^

The concept of infectious burden (IB)—defined as the cumulative serological evidence of exposure to various prevalent pathogens—is gaining recognition for its potential impact on health.^47^ Analogous to infections with individual pathogens, an increasing number of studies have found a correlation between IB and vascular diseases. One finding indicates significant differences in IB between groups with and without CMBs.^48,49^ Notably, an elevated IB correlates independently with the presence of CMBs and shows a positive association with the number of CMBs.^50^ In a retrospective cohort study, it was observed that 18% of patients with central nervous system infections developed CMBs within one year following the initial infection—a rate 47 times higher than that observed in controls without central nervous system (CNS) infections.^51^ Furthermore, case studies from various countries have documented CMBs across a diverse age range in association with atopic pathogenic infections, including spotted fever rickettsia and ehrlichiosis.^52^

De Stefano *et al*. described a case involving a 56-year-old female smoker with emphysema and hypothyroidism, who developed critical illness-related CMBs after a COVID-19 diagnosis, characterized by focal changes on electroencephalography. This case represents the first documented instance of CMBs linked to severe acute respiratory syndrome coronavirus 2 infection.^53^ Nonetheless, the occurrence of CMBs in the context of SARS-CoV-2 is not merely coincidental. Bryce *et al*. reported the presence of extensive microthrombosis and acute infarctions in the brain tissue of deceased COVID-19 patients (30%), with some infarcted regions also exhibiting acute parenchymal CMBs.^54^ These findings raise the possibility of direct viral invasion, supported by the detection of viral RNA and proteins in the brains of COVID-19 patients who had neurological manifestations, thereby implying viral infiltration of the human brain.^26^ However, this evidence alone does not conclusively establish that CMBs are directly caused by SARS-CoV-2 invading neurons or blood vessels.

Current research reveals a complex link between COVID-19 and CMBs, with age and vascular risks notably affecting CMBs rates. The direct impact of COVID-19 on CMBs development is still unclear, highlighting the need for detailed investigations to clarify any direct connections. Variations in CMBs incidence call for standardized data gathering and analysis to better comprehend this aspect of COVID-19. Additionally, the effect of infection on vascular health, including CMBs, must be factored into COVID-19’s overall health assessments. To address these challenges, it is critical to update clinical guidelines to incorporate these findings, improving risk assessment and treatment strategies for cerebrovascular diseases amid the pandemic. Such revisions are crucial for maintaining evidence-based practices and improving patient outcomes concerning COVID-19-associated cerebrovascular issues.

## Brain pathology and clinical consequence of CMBs in COVID-19

Among patients with mild to moderate respiratory illness, neurological manifestations tend to be nonspecific, ranging from headaches and dizziness to more distinct yet non-debilitating symptoms like hypotension.^5^ In contrast, severe respiratory conditions often correlate with pronounced neurological deficits and altered consciousness. These severe presentations may stem from acute toxic encephalitis—attributable to systemic toxaemia, metabolic imbalances and hypoxia—or from inflammatory cerebrovascular damage, which is closely linked to the onset of systemic inflammatory response syndrome.^55,56^

A synthesis of the current literature reveals that CMBs after COVID-19 infection may manifest with a range of neurological symptoms, from agitation and delirium to more severe disturbances such as acute changes in consciousness, aphasia or quadriparesis.^53,57,58^ CMBs are particularly prevalent in the brain tissue of patients in critical condition, notably those with acute respiratory distress or disseminated intravascular coagulation.^59^ These microbleeds are frequently observed within the subcortical white matter and corpus callosum in patients enduring hypoxaemia and extended respiratory failure^60^ (**Table 2**). Notably, recent findings indicate that CMBs can also arise in patients experiencing only mild COVID-19 symptoms, with a significantly elevated occurrence in those who concurrently suffer from acute stroke.^61^

**Table 2:** Anatomic location and clinical characteristics of micro bleeding after COVID-19

Study	Test method	Number of respondents	Body parts	Neuro-clinical manifestations	Title of thesis
Linda Backman	MRI, T2WI, T1WI, FLAIR, SWI, and DWI	1	The right temporal lobe, left basal ganglia, and right cerebellar hemisphere	Disoriented, cognitive deficits, delirious, visual hallucinations, and severe sleeping disorders	(28)
Pia De Stefano	MRI and SWI	1	Bilateral juxtacortical white matter, corpus callosum, and internal capsule	Persistent slight executive dysfunction	(51)
Daniel Kirschenbaum	MRI and SWI	4	Juxtacortical microbleeds, most conspicuous in the frontal lobe	Confusion at admission, asymmetric reactive pupils, and negative wake-up	(54)
Aikaterini Fitsiori	MRI, T2WI, T1WI, FLAIR, and SWI	9	Corpus callosum, the internal capsule (5/9), and middle cerebellar peduncles (5/9)	Agitation or delayed recovery of consciousness, a withdrawal syndrome from sedation and benzodiazepines	(55)
Pedro Fraiman	GRE T2-weighted MRI	1	Cerebral lobes and cerebellum	Acute impairment of cinsciousness	(56)
Khawaja Hassan Haroon	MRI and SWI	1	In the bilateral cortical-juxtacortical regions, deep white matter, basal ganglia, the corpus callosum, the brain stem, and the cerebellum	Delirious	(74)
Juan Montes-Ramirez	MRI and SWI	1	Corpus callosum	Aphasia and quadriparesia	(76)
Myoung-Hwa Lee	HR-MRI	9	Olfactory bulb and brain stem	Delirium	(99)
Dioselina Panamá Tristán-Samaniego	Brain CT, FLAIR MRI, DWI, and SWI	1	Supratentorial space	Reduced consciousness, mixed delirium with marked disorientation, mild agitation, slow thoughts and speech, right central facial paralysis, and generalized hyperreflexia	(110)

Abbreviations: (1) MRI: magnetic resonance imaging; (2) SWI: susceptibility-weighted imaging; (3) FLAIR: fluid attenuated inversion recovery; (4) GRE T2-weighted MRI: gradient-recalled echo T2-weighted imaging; (5) HR-MRI: high-resolution MRI; (6) CT: computed tomography; (7) DWI: diffusion weighted imaging.

### Behavioural manifestations: common cognitive impairment

In a case study by Fraiman *et al*., a 38-year-old female patient without prior cardiovascular risk factors was admitted for COVID-19 and exhibited profound consciousness impairment. She demonstrated global aphasia, mobility difficulties, myoclonic jerks and intermittent generalized seizures. T2-weighted axial gradient echo MRI scans identified dispersed microhaemorrhages across the cerebral lobes and cerebellum.^58^ Separately, a 45-year-old male, following a severe progression of COVID-19 marked by acute respiratory distress syndrome (ARDS) and CMBs, required 35 days of mechanical ventilation, starting approximately 10 days post-infection onset. At a 3-month follow-up, the patient is presented with mild to moderate impairments in processing speed, working memory and attention. At 8 months, persistent mild impairments were noted in logical reasoning, attention, executive functions and processing speed.^28^ These cases underscore the potential for enduring cognitive deficits following severe COVID-19, underscoring the importance of research into the cognitive aftermath and cerebrovascular damage linked to the disease.

Cognitive dysfunction is more prevalent among patients with CMBs. The behavioural impact of CMBs appears to be influenced by several factors, such as the quantity, size and location of the CMBs, as well as the presence of comorbidities.^62^ Evidence suggests a correlation between a higher burden of CMBs and more pronounced cognitive deficits.^42^ Cognitive assessments reveal that the presence of CMBs is linked to compromised executive function, diminished attention, slower processing speeds and general cognitive decline.^63^ The underlying mechanisms posited for CMB-related cognitive impairment include localized brain damage and the subsequent disruption of communication between neurons and astrocytes in surrounding areas.^64^ Recent research indicates that accumulations of CMBs may disrupt the structural connectivity of the brain.^65^ Bergeron *et al*. introduced a novel mouse model for studying CMBs through cortical collagenase injection and observed a significant decline in learning, spatial and visuospatial memory in mice with CMBs at six weeks post-injection. Interestingly, atorvastatin treatment was found to markedly enhance cognitive function in these mice.^65^ In the context of COVID-19, patients have been reported to experience a spectrum of enduring symptoms, including cognitive and psychological issues.^66,67^ However, one study highlighted that while survivors of COVID-19 from intensive care units exhibited a higher incidence of microbleeds, they did not show a greater prevalence of cognitive dysfunction when compared to patients who recovered in general wards.^68^

Despite progress in understanding CMBs and their cognitive implications, several constraints hinder the precise correlation between CMBs locations and cognitive outcomes. Limitations encompass the imaging techniques employed, the demographic diversity of study populations, confounding factors that may influence performance on standard cognitive assessments and the relatively limited sensitivity of current cognitive testing methods.

### White matter lesions: a bad indicator

Recently, critical illness-related CMBs have been increasingly observed in patients with respiratory failure requiring mechanical ventilation, particularly those with systemic illnesses.^69^ MRI studies demonstrate a significant correlation between the quantity of CMBs and the severity of white matter lesions.^17^ COVID-19-related small vessel disease tends to affect the subcortical white matter, corpus callosum, juxtacortical regions and periventricular areas.^56,70^ The present study indicates that a substantial subset of COVID-19 patients exhibit neurological abnormalities detectable on MRI, with the most frequent alterations being changes in perfusion, white matter integrity, evidence of acute or subacute strokes, olfactory bulb changes and CMBs.^71,72^ Motor abnormalities such as heightened tendon reflexes, ankle clonus and bilateral Babinski signs are commonly associated with these white matter changes.^73^ This is primarily due to the involvement of the pyramidal tract—the principal white matter conduit linking key subcortical structures like the basal ganglia and thalamus—which plays a vital role in the coordination of voluntary motor control.^74,75^

Also, the association of white matter encephalopathy and CMBs with critical illness, higher mortality rates and functional decline has been documented in COVID-19 patients.^76^ In a retrospective study by Agarwal *et al*., which included 4131 adults with COVID-19 in New York City, MRI evaluations of 115 patients revealed that 35 (30.4%) exhibited signs of leukoencephalopathy and/or CMBs.^19^ Imaging studies suggest small vessel disease as the underlying cause of white matter damage in leukoencephalopathy, with CMBs increasingly recognized for their role in the development and functional impact of this condition.^77,78^

Current evidence from neuropathological examinations suggests that patients with diffuse white matter brain diseases exhibit compromised integrity of the blood–brain barrier.^79^ We propose that endothelial cell activation, potentially triggered by COVID-19, may breach the barrier, facilitating the influx of inflammatory mediators and neurotoxic factors that contribute to the development of white matter diseases. Furthermore, these patients often endure severe conditions, including prolonged mechanical ventilation and moderate to severe ARDS, in intensive care settings. This situation may result in cerebral hypoxia and ischaemic damage stemming from persistent shock and refractory hypoxia, potentially leading to conditions akin to delayed posthypoxic leukoencephalopathy. There is a pressing need for further neuropathological studies to elucidate the tissue and molecular mechanisms underlying such injuries in these patients.

### Characteristics of imaging: an important diagnostic basis

In a retrospective analysis examining brain MRI findings in patients with COVID-19 and concurrent nonischaemic cerebral infarction, 9 out of 37 cases displayed extensive white matter microhaemorrhages.^8^ CMBs are visualized on MRI as hypointense spots, especially with T2-weighted or susceptibility-weighted sequences.^64^ The imaging characteristics of CMBs stem from localized variations in magnetic properties due to the accumulation of iron, often within perivascular macrophages following vascular injury.^80^ These macrophages metabolize haemoglobin from erythrocytes, leading to the deposition of paramagnetic iron, which is detectable with T2*-weighted gradient-recalled echo sequences.^10^ Differentiation of CMBs from other entities such as vascular flow voids, bone or deposits of iron or calcium is critical, and the current guidelines suggest using a maximum diameter of 5–10 mm to reliably distinguish CMBs from larger haemorrhages.^9,11^

The recognition of CMBs has surged since the early 1990s, aided by the advent of MRI techniques.^6,9^ As research has advanced, detection accuracy for CMBs using MRI at 1.5 Tesla (T) or 3.0 T, particularly with T2*-weighted or susceptibility-weighted sequences, ranges between 48% and 89%.^6,81^ However, it is notable that clinical MRI can miss over half of the CMBs that are later identified through postmortem histopathological analysis. Enhancements in MRI, such as magnetization rate-weighted imaging, have significantly increased the detection rates for certain pathologies, such as giant cell tumours, with accuracies up to 98.1%.^82^ Despite these advancements, MRI remains limited compared to histopathological examination, with challenges such as imaging artefacts and the inability to definitively identify specific tissue types or pinpoint microvascular damage.^83^

In the progression of COVID-19, CMBs appear to be a late-stage event in the cascade of disease manifestations. Early diagnostic approaches, such as the use of biomarkers, could significantly aid in predicting outcomes, thereby facilitating adjustments in treatment strategies for affected patients. Clinical research has demonstrated that hospitalized COVID-19 patients frequently exhibit CMBs accompanied by elevated levels of inflammatory markers including C-reactive protein, procalcitonin, tumour necrosis factor alpha and soluble interleukin-2 receptor.^84^ Additionally, there is a notable presence of consumptive coagulopathy in COVID-19 patients, characterized by increased levels of D-dimer and fibrinogen degradation products. This condition may lead to the formation of small medullary vein thromboses, subsequently causing CMBs.^85^ Another study highlighted that patient with CMBs had lower platelet counts and higher D-dimer levels, supporting this association.^19^ Investigations involving glial fibrillary acidic protein, neurofilament light chain and tau protein have also identified correlations with CMBs^86-88^; however, further studies are necessary to confirm these findings in COVID-19 patients. Exploring blood biomarkers will provide more insights into the pathophysiology and mechanisms underlying COVID-19-associated microbleeds, with many mechanisms still to be elucidated.

## Pathogenesis

**Figure 1:**
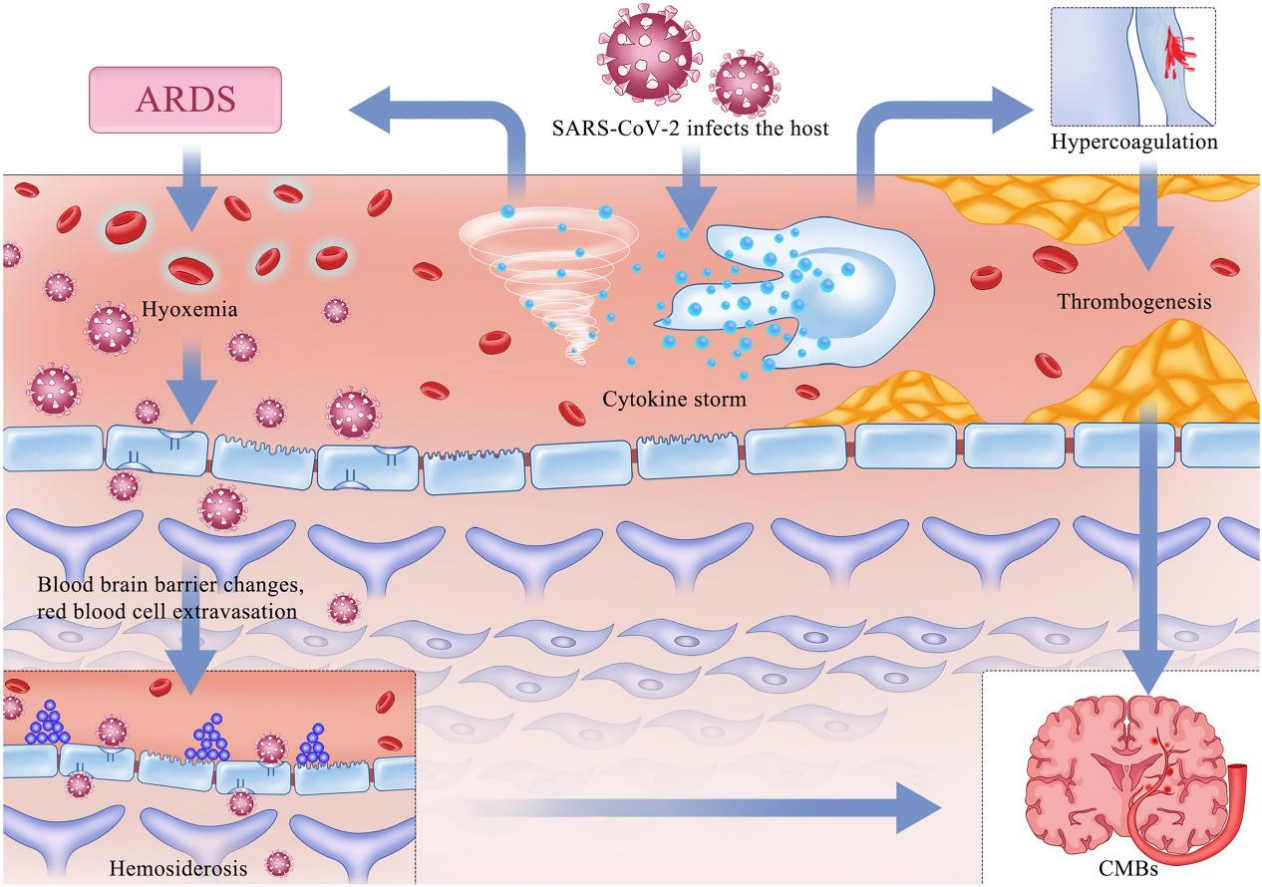
**The formation mechanism of CMBs in patients with COVID-19.** SARS-CoV-2 enters the host by binding to the ACE2 receptor on lung cells, leading to endothelial injury. This damage prompts a release of cytokines from endothelial and immune cells, potentially resulting in a cytokine storm. The consequent inflammatory response elevates the risk of ARDS, which impairs oxygenation of the blood, a condition known as hypoxaemia. Hypoxaemia can disrupt the blood–brain barrier through stasis or biochemical reactions, permitting red blood cells to escape into the brain tissue and deposit hemosiderin. Concurrently, cytokine storms can damage the vascular endothelium and disrupt the coagulation system, increasing the propensity for thrombus formation. Such thrombi can compromise cerebral vascular function and contribute to the development of CMBs. Abbreviations: severe acute respiratory syndrome coronavirus 2(SARS-CoV-2), coronavirus disease 2019 (COVID-19), angiotensinogen-converting enzyme 2(ACE2), acute respiratory distress syndrome (ARDS), cerebral microbleeds (CMBs).

The aetiology of CMBs following COVID-19 infection remains uncertain; however, data from previous studies suggest cerebrovascular inflammation as a primary mechanism. This inflammation may stem from heightened cytokine levels secondary to COVID-19 or from endothelial dysfunction within a pro-coagulant environment.^89^ A consistent finding among affected patients is severe hypoxaemia due to ARDS, necessitating extended mechanical ventilation and the use of a spectrum of antiviral and antibiotic medications.^56^ Such hypoxaemia may induce hydrostatic or chemical disruptions to the blood–brain barrier, leading to the leakage of red blood cells into the brain parenchyma^69^ (Fig. 1).

### Systemic inflammatory response

Cytokine dysregulation has been implicated in central nervous system complications associated with COVID-19.^89^ An extensive study involving 67 COVID-19-positive patients, which included complete autopsies with thorough microscopic examination, transmission electron microscopy, immunohistochemistry, RNA in situ hybridization, immunologic assays and serology, revealed heightened levels of inflammatory markers. Notably, increased concentrations of cytokines such as IL-6, IL-8 and tumor necrosis factor-α (TNF-α) were observed alongside evidence of hemophagocytosis and phagocytic lymph histiocytosis.^54^ This constellation of findings points to microangiopathy and a hyperinflammatory state as potential contributors to the development of CMBs.^90^ Building on this, recent clinical research has posited a relationship between systemic inflammation and CMBs pathogenesis. Corroborative experimental work has been conducted, with researchers establishing animal models of CMBs induced by lipopolysaccharide—a standardized inflammatory agent—thereby providing a basis for the direct causative role of inflammation in the genesis of CMBs.^91^

The inflammatory cascade is a critical factor in the cognitive deterioration seen subsequent to CMBs. Post-CMBs, the inflammatory response encompasses the infiltration of leukocytes from the bloodstream, the migration and proliferation of microglia and the activation of astrocytes. These processes collectively disrupt the surrounding neural milieu and compromise neuronal function.^92^ A comprehensive MRI analysis coupled with quantitative evaluation has linked CMBs presence to inflammatory markers in the cerebrospinal fluid. Notably, there is an association between CMBs and decreased haemoglobin and lymphocyte levels, as well as elevated counts of leukocytes, procalcitonin, lactate dehydrogenase and C-reactive protein. These markers correlate with the total volume of CMBs.^29^ Recent experimental advancements suggest that the deletion of myosin light chain kinase (MLCK) confers protection against CMBs and the cerebral effects of chronic dietary neuroinflammation, such as hyperhomocysteinemia.^93^ This discovery indicates that MLCK inhibition may represent a novel therapeutic avenue for combating tissue barrier dysfunctions, especially blood–brain barrier compromises, and could provide benefits in neuroinflammation-related models of vascular cognitive impairment and dementia.^94^

### Vascular endothelial response

In an initial report by Kirschenbaum *et al*., two patients with confirmed SARS-CoV-2 infections displayed signs of cerebral endothelial infection. Neuropathological evaluations revealed that the ACE2 receptor, which SARS-CoV-2 utilizes for entry, is indeed present in the cerebral endothelium, suggesting a potential direct effect of the virus on these cells.^8^ While ACE2 receptors are the primary means of viral entry in the lungs,^95^ they are also present in various extrapulmonary sites, including the heart, kidneys and central nervous system, with vascular endothelial cells expressing these receptors as well. However, the expression of ACE2 in neuronal and astrocytic cells within the CNS is notably low compared to that in pulmonary tissues.^96-98^ However, the expression of ACE2 in neuronal and astrocytic cells within the CNS is notably low compared to that in pulmonary tissues. In contrast, the neuropilin-1 receptor, another facilitator of SARS-CoV-2 infection, is abundantly expressed in neurons and astrocytes, particularly within the olfactory epithelium and CNS.^99,100^ Recent observations have highlighted multifocal microvascular damage in the brain and olfactory bulb.^101^ Histopathological examinations have noted the following: a thinning of the endothelial cell basal layer, areas of linear low signal on imaging suggestive of microbleeds, and in some cases, the presence of perivascular activated microglia, macrophage infiltration and hypertrophic astrocytes. Magnetic resonance microscopy has revealed focal high signals consistent with microvascular injury and fibrinogen extravasation.^101^ The extent to which SARS-CoV-2 contributes to vascular pathology in COVID-19 warrants further investigation in our ongoing study.

Epidemiological studies have established that the elderly constitute the majority of those infected with COVID-19, suggesting that the age-related vascular changes, including oxidative stress and endothelial dysfunction, may contribute to the occurrence of CMBs in this age group.^102-104^ Hypertension exacerbates this risk by promoting oxidative stress and activating matrix metalloproteinases, which lead to the degradation of the vascular extracellular matrix.^105^ CMBs resulting from hypertensive vasculopathy typically involve the small penetrating arteries of the deep brain regions, including the basal ganglia, brainstem, cerebellum and white matter.^106^ This pattern is different from the CMBs observed post-COVID-19. Research using mouse models has shown that the aging process can intensify the effect of hypertension on oxidative stress and matrix metalloproteinase (MMP) activity, thereby increasing the formation of CMBs.^107^ Activation of MMPs has been implicated in a range of pathophysiological conditions such as CAA-related haemorrhage, Alzheimer’s disease and hyperhomocysteinemia, suggesting a potential shared pathway in the development of CMBs.^108,109^ MMPs compromise the structural integrity of cerebral vessels by degrading key elements like collagen and elastin, critical to maintaining vascular stability.^24^

### Hypoxaemia

CMBs have been observed in the subcortical white matter and corpus callosum of critically ill patients, particularly those suffering from hypoxaemia and prolonged respiratory failure.^8,110^ The pathogenesis of these CMBs shares similarities with those seen in patients exposed to sustained hypoxaemia, such as at high altitudes, but is not identical.^12^ A study in 2017 documented extensive microbleeds on MRI scans in 12 intensive care unit patients who experienced respiratory failure; 11 of these patients were mechanically ventilated. These microbleeds predominantly affected the subcortical white matter and were less prevalent in the cortex, deep white matter, basal ganglia and thalamus.^69^ Notably, the patients were significantly younger than those typically presenting with hypertension-related CMBs or sporadic CAA, and hypoxia was the primary cause of death.^69^ Moreover, histopathological analysis of the brains from 18 patients who succumbed to COVID-19 revealed only hypoxic changes, with no evidence of encephalitis or other virus-specific brain pathologies.^27^ Observational studies have found that the CMB burden remains stable over time, suggesting a possible link to severe hypoxaemia rather than transient factors.^23,111^ In a case report, a 46-year-old male patient with COVID-19 showed a significant resolution of white matter lesions on MRI over a 23-day post-discharge period, with no progression in microbleed burden. This case adds to the mounting evidence that COVID-19 may increase the risk of microbleeds, potentially due to hypoxaemic injury rather than direct viral effects on the cerebral vasculature.^112^

Radmanesh *et al*. suggested diffuse leukoencephalopathy and CMBs as potential late sequelae in patients with COVID-19 who experienced severe hypoxaemia.^22^ The pathogenesis of CMBs under these conditions remains elusive; however, it is postulated that the microvascular damage and subacute inflammatory responses may compromise the blood–brain barrier. In a study, 30% of COVID-19 patients who exhibited CMBs demonstrated elevated levels of Immunoglobulin G and an increased albumin quotient following lumbar puncture, consistent with disruption of the blood–brain barrier.^23^ This disruption, coupled with neurovascular unit dysfunction, could lead to endothelial impairment, haemorrhage and the subsequent deposition of haeme products.^113^ Furthermore, the occurrence of CMBs within the corpus callosum in COVID-19 patients has been linked to the effects of mechanical ventilation, rather than a direct viral invasion.^110,114^ Reports indicate that the majority of COVID-19 fatalities involved ARDS and subsequent mechanical ventilation.^115,116^ Additionally, diffuse microbleeds have been posited as a complication of extracorporeal membrane oxygenation in the context of COVID-19, despite being previously considered an infrequent adverse event associated with extracorporeal membrane oxygenation (ECMO) therapy.^117,118^

While a causal link has been suggested between cerebral haemorrhage, SARS-CoV-2-induced endothelial inflammation and hypoxaemic injury, the predominantly retrospective nature of existing studies and their reliance on small patient cohorts limit the robustness of these conclusions. Consequently, there is a clear need for more extensive, prospective research to validate these findings.

## Conclusions

The current body of research on CMBs post-COVID-19 infection has largely been epidemiological. This review represents a pioneering, comprehensive examination of the latest developments in the epidemiology, neuropathology, clinical implications and pathophysiology of CMBs in the context of COVID-19. CMBs associated with SARS-CoV-2 are more than mere coincidences; they exhibit a significant correlation with the infection. Emerging imaging evidence indicates that CMBs, particularly in the proximal cortical regions and corpus callosum, commonly occur alongside white matter pathology and are often accompanied by cognitive impairments in COVID-19 patients. Inflammatory responses within the cerebrovasculature, characterized by elevated cytokine levels post-infection or endothelial dysfunction amid a pro-coagulant state, are central to this phenomenon. A consistent finding across affected individuals is profound hypoxaemia, which may induce hydrostatic or chemical disruptions to the blood–brain barrier, leading to red blood cell extravasation. Despite their clinical significance, the exact mechanisms precipitating the observed increase in CMBs incidence post-COVID-19 remain elusive in human studies. Presently, no effective preventative strategies have been established. To elucidate these mechanisms and develop interventions, further basic and clinical prospective research is imperative.

## Data Availability

The datasets used and analysed during the current study are available from the corresponding author upon reasonable request.
